# Antipyrin as a Uterine Sedative

**Published:** 1888-04

**Authors:** 


ANTIPYRIN AS A UTERINE SEDATIVE.—M. H. Chouppe has already called attention to the good effects of antipyrin in uterine pains after parturition or in dysmenorrhœa. In proof of this, he relates the following case: A woman, aged thirty-five, was suffering from a large myoma, situated in the posterior wall of the uterus, accompanied by copious hemorrhage, which reappeared after menstruation. Ergot checked the hemorrhage, but caused such severe uterine pains that it had to be discontinued. Large doses of morphine were administered; these caused the pains to disappear, but at the same time the uterine contraction was relaxed and hemorrhage reappeared. Ergot was again administered, with similar result. The attacks of uterine pains lasted two or three hours. M. Chouppe then had recourse to antipyrin. An injection, containing two grammes of antipyrin, was administered. At the end of twenty minutes, the pain disappeared. M. Chouppe then tried the following experiment: An injection of antipyrin was given half an hour before the dose of ergot. The patient experienced no pain, although there was active uterine contraction; hemorrhage was arrested. M. Chouppe concludes that antipyrin relieves the pain caused by the contraction which is produced by ergot without diminishing the contraction. He believes that it acts upon the spinal cord, and might be administered with advantage during parturition to women of an irritable temperament.—*Brit. Med. Jour.*

